# Pentastatin, a matrikine of the collagen IVα5, is a novel endogenous mediator of pulmonary endothelial dysfunction

**DOI:** 10.1152/ajpcell.00391.2023

**Published:** 2023-09-11

**Authors:** Ayse Ceren Mutgan, Katharina Jandl, Nemanja Radic, Francesco Valzano, Dagmar Kolb, Julia Hoffmann, Vasile Foris, Jochen Wilhelm, Panja M. Boehm, Konrad Hoetzenecker, Andrea Olschewski, Horst Olschewski, Akos Heinemann, Malgorzata Wygrecka, Leigh M. Marsh, Grazyna Kwapiszewska

**Affiliations:** ^1^Division of Physiology and Pathophysiology, Otto Loewi Research Center, Medical University of Graz, Graz, Austria; ^2^Ludwig Boltzmann Institute for Lung Vascular Research, Graz, Austria; ^3^Division of Pharmacology, Otto Loewi Research Center, Medical University of Graz, Graz, Austria; ^4^Core Facility Ultrastructure Analysis, Center for Medical Research, Medical University of Graz, Graz, Austria; ^5^Division of Pulmonology, Medical University of Graz, Graz, Austria; ^6^Institute for Lung Health, Member of the German Lung Center (DZL), Giessen, Germany; ^7^Department of Thoracic Surgery, Medical University of Vienna, Vienna, Austria; ^8^Department of Anaesthesiology and Intensive Care Medicine, Medical University of Graz, Graz, Austria; ^9^Center for Infection and Genomics of the Lung, Universities of Giessen and Marburg Lung Center, Giessen, Germany

**Keywords:** collagen IVα5, endothelial dysfunction, matrikine, pentastatin, pulmonary hypertension

## Abstract

Deposition of basement membrane components, such as collagen IVα5, is associated with altered endothelial cell function in pulmonary hypertension. Collagen IVα5 harbors a functionally active fragment within its C-terminal noncollageneous (NC1) domain, called pentastatin, whose role in pulmonary endothelial cell behavior remains unknown. Here, we demonstrate that pentastatin serves as a mediator of pulmonary endothelial cell dysfunction, contributing to pulmonary hypertension. In vitro, treatment with pentastatin induced transcription of immediate early genes and proinflammatory cytokines and led to a functional loss of endothelial barrier integrity in pulmonary arterial endothelial cells. Mechanistically, pentastatin leads to β1-integrin subunit clustering and Rho/ROCK activation. Blockage of the β1-integrin subunit or the Rho/ROCK pathway partially attenuated the pentastatin-induced endothelial barrier disruption. Although pentastatin reduced the viability of endothelial cells, smooth muscle cell proliferation was induced. These effects on the pulmonary vascular cells were recapitulated ex vivo in the isolated-perfused lung model, where treatment with pentastatin-induced swelling of the endothelium accompanied by occasional endothelial cell apoptosis. This was reflected by increased vascular permeability and elevated pulmonary arterial pressure induced by pentastatin. This study identifies pentastatin as a mediator of endothelial cell dysfunction, which thus might contribute to the pathogenesis of pulmonary vascular disorders such as pulmonary hypertension.

**NEW & NOTEWORTHY** This study is the first to show that pentastatin, the matrikine of the basement membrane (BM) collagen IVα5 polypeptide, triggers rapid pulmonary arterial endothelial cell barrier disruption, activation, and apoptosis in vitro and ex vivo. Mechanistically, pentastatin partially acts through binding to the β1-integrin subunit and the Rho/ROCK pathway. These findings are the first to link pentastatin to pulmonary endothelial dysfunction and, thus, suggest a major role for BM-matrikines in pulmonary vascular diseases such as pulmonary hypertension.

## Graphical abstract

**Figure:**
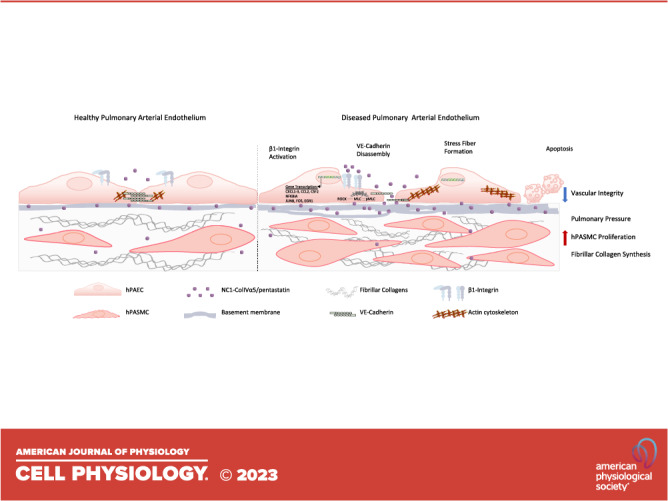

## INTRODUCTION

The basement membrane (BM) has increasingly emerged as an important determinant of endothelial cell (EC) function, thereby contributing to pulmonary vascular diseases such as pulmonary hypertension (PH) (1). The BM consists of a set of extracellular matrix (ECM) proteins, mainly type IV collagens, laminins, and proteoglycans such as perlecan and collagen XVIIIα1 (ColXVIIIα1) (2, 3). Previously, we observed a substantial increase in gene expression of genes encoding several BM proteins and polypeptides, including collagen IVα5 (ColIVα5) and ColXVIIIα1, in remodeled pulmonary arteries (PAs) of patients with idiopathic pulmonary arterial hypertension (IPAH) (1, 4). Furthermore, BM remodeling was characterized by regions of BM thickening and degradation (1).

In remodeled vessels, the combination of increased immune cell numbers (5, 6) and protease activity (3, 7) can lead to the fragmentation of BM and, thus, a rise in bioactive matrikine fragments. Indeed, elevated plasma levels of endostatin, the matrikine of ColXVIIIα1, were reported in patients with IPAH and correlated with disease severity and mortality (4, 8). Functionally, this was linked to decreased endothelial cell survival (9).

Similarly, bioactive matrikine sequences are found in the C-terminal noncollagenous (NC1) domains of the individual type IV collagen polypeptides (α1 to α6) (10). Those unique NC1-derived matrikines potently, yet differentially, alter endothelial cell function and angiogenesis. For example, an anti-angiogenic potential was identified for the recombinant sequence of the α1, α2, α3, and α6(IV)NC1 domains (11–15), while little to no effect was identified with α4 and α5(IV)NC1 domains (11). Interestingly, within the NC1 domain of collagen IVα5, a 20 amino acid-long bioactive sequence, termed pentastatin, has been predicted to possess anti-angiogenic potential (16, 17). Several in vitro studies, mostly using human umbilical vein endothelial cells (HUVECs) (16, 17) and some in vivo studies, conducting microcapillary experiments in mice and using tumor xenograft mouse models, have validated this potential (18, 19). Besides angiogenesis, endothelial cell barrier integrity is a crucial hallmark of proper endothelial cell function and vascular homeostasis. Thus, the alteration of endothelial barrier function is associated with various pulmonary vascular diseases, such as pulmonary hypertension.

Here, we are first to demonstrate both in vitro and ex vivo how exogenous treatment with the ColIVα5 NC1 fragment (NC1-ColIVα5) and its shorter peptide, pentastatin, impairs endothelial cell function by inducing apoptosis and barrier disruption. This was partially mediated by the β1-integrin subunit and Rho/ROCK signaling. Ex vivo, pentastatin increased mean pulmonary arterial pressure (mPAP) with a concomitant decrease in vascular integrity. Herewith, this study proposes that pentastatin is a potent factor that sustains a dysfunctional endothelium and thus may contribute to the pathogenesis of pulmonary vascular diseases, such as PH.

## MATERIAL AND METHODS

### Human Samples

Human lung tissues were obtained from patients with end-stage IPAH who underwent lung transplantation at the Department of Thoracic Surgery, Medical University of Vienna, Austria. Nontransplanted, nontumorous, downsized healthy lungs were used as controls. The institutional ethics committee approved the protocol and tissue usage (976/2010), and patient consent was obtained before lung transplantation. Human pulmonary arterial endothelial cells (hPAECs) and human pulmonary smooth muscle cells (hPASMCs) were isolated freshly, as previously described (20), from the pulmonary arteries of explanted lung tissues provided by the Department of Thoracic Surgery, Medical University of Vienna.

Plasma from patients with end-stage IPAH and matched non-PH comparators were obtained from the outpatients’ clinic. The ethics committee of the Medical University of Graz approved the protocol (23–408 ex 10/11), and the patient consent was received before the blood collection.

### Pulmonary Arterial Endothelial and Smooth Muscle Cell Culture

For in vitro experiments, hPAECs from healthy individuals were either purchased (Lonza, Basel, Switzerland) or isolated according to the in-house protocol (20). For gene expression analysis, hPAECs isolated from patients with end-stage IPAH were used. hPAECs were maintained in full endothelial cell media; EBM-2 endothelial cell growth basal medium (Lonza, Basel, Switzerland) with EGM-2 endothelial SingleQuots kit (Lonza, Basel, Switzerland) on precoated culture dishes with 1% (m/v) gelatin (Merck Group, Darmstadt, Germany). hPASMCs were cultured in VascuLife SMC Medium Complete Kit (Lifeline Cell Technology, California). hPAECs between passages 3 and 8 and hPASMCs between passages 2 and 3 were used for experiments. Information on hPAECs and hPASMCs used in this study is provided in Table 1.

**Table 1.** Information on the primary cells used in the study

Cell ID	Cell Type	Age, yr	Sex, F/M	Diagnosis
hPAEC1	hPAEC	45	M	Healthy
hPAEC2	hPAEC	63	M	Healthy
hPAEC3	hPAEC	59	M	Healthy
hPAEC4	hPAEC	21	M	Healthy
hPAEC5	hPAEC	45	F	Healthy
hPAEC6	hPAEC	21	M	Healthy
hPAEC7	hPAEC	51	M	Healthy
hPAEC8	hPAEC	52	F	Healthy
hPAEC9	hPAEC	48	M	Healthy
hPAEC10	hPAEC	33	F	Healthy
hPAEC11	hPAEC	42	M	Healthy
hPAEC12	hPAEC	60	M	Healthy
hPAEC13	hPAEC	59	F	Healthy
IPAH-hPAEC1	hPAEC	41	F	IPAH
IPAH-hPAEC2	hPAEC	21	M	IPAH
IPAH-hPAEC3	hPAEC	37	M	IPAH
IPAH-hPAEC4	hPAEC	38	F	IPAH
hPASMC1	hPASMC	32	M	Healthy
hPASMC2	hPASMC	56	M	Healthy
hPASMC3	hPASMC	31	M	Healthy
hPASMC4	hPASMC	59	M	Healthy
hPASMC5	hPASMC	58	M	Healthy
hPASMC6	hPASMC	57	F	Healthy

F/M, female/male; hPAEC, human pulmonary endothelial cell; hPASMC, human pulmonary smooth muscle cell.

### Cell Culture Stimulations of hPAECs and hPASMCs

In vitro experiments with the recombinant C-terminal NC1 fragment (Gly1461 ∼ Thr1685, NC1, RPC141Hu01, Cloud-Clone, Wuhan, China) of the collagen IVα5 polypeptide, pentastatin (PS, LRRFSTMPFMFCNINNVCNF), random peptide control (RP, YGRKKRRQRRRGGNWAWHDFVHIT) (PS and RP both from Biomatik, Ontario, Canada, and PANATecs, Heilbronn, Germany), or vehicle [veh, dimethyl sulfoxide (DMSO); AppliChem, Darmstadt, Germany] were performed on confluent monolayers of hPAECs and hPASMCs at 70–80% confluency. Pentastatin and random peptide control were reconstituted in 100% (m/v) DMSO to reach 10 mg/mL stock solution. For all experiments, 0.5% (w/v) DMSO, which is equal to the volume of the highest pentastatin/random peptide concentration (50 µg/mL), was used. The endotoxin levels in purchased synthetic peptides, namely pentastatin and random peptide control, were measured by using Limulus amebocyte lysate (LAL) assay-based Pierce Chromogenic Endotoxin Quant Kit (#A39552, Thermo Fisher Scientific, Massachusetts) according to the manufacturer’s instructions.

Thrombin (#T4648, Sigma-Aldrich, Missouri) was used as a positive control to assess a decrease in endothelial resistance. To pharmacologically block Rho/ROCK pathway and β1-integrin, hPAECs were pretreated with the ROCK inhibitor Y-27632 (#10005583, Cayman Chemical Company, Michigan) and an anti-β1-integrin subunit antibody (#MAB17781, clone P5D2, R&D Systems, Minnesota).

In addition, hPAECs were stimulated with VEGF (#293-VE, R&D Systems, Minnesota) and TGF-β (240-B, R&D Systems, Minnesota) at 70–80% confluency for 24 h.

### Monitoring Endothelial Barrier Function

The endothelial barrier function of hPAECs was monitored using the electrical cell-substrate impedance sensing system (ECIS, Applied Biophysics, New York). hPAECs were seeded at the density of 150,000 cells per well in full endothelial cell media on ECIS cell culture chips (8W10E+ PET chips, Applied Biophysics, New York) preactivated with 10 mM l-cysteine (Sigma-Aldrich, Missouri) and precoated with 1% (m/v) gelatin. Chips were then transferred to the ECIS system (ECIS Z-Theta, Applied Biophysics, New York) to monitor real-time barrier integrity by measuring electrical resistance. Endothelial cells, including stimulations, were maintained in full endothelial cell media throughout the experiment. Electrical resistance was recorded every 90 s for each well. After establishing a stable endothelial barrier, a baseline resistance measurement was recorded 2 h before the experiment. Consequently, hPAECs were treated with vehicle, NC1, pentastatin, or random peptide. Endothelial cell resistance was monitored for at least 20 h posttreatment to assess changes in endothelial barrier integrity.

To evaluate the reversibility of the pentastatin effect, recovery experiments were performed on hPAECs using ECIS. Five hours after pentastatin exposure in full endothelial cell media, media in wells were replaced with fresh full endothelial cell media without stimulants and monitored for at least 20 h.

At the end of the experiments, endothelial resistance at 4,000 hertz was analyzed. Absolute values for resistance at any given point were normalized to the average absolute resistance at the baseline. Data is presented as a plot of normalized resistance versus time. Changes are displayed as the percentage of the normalized resistance at the baseline.

### Immunofluorescence Staining of hPAECs

hPAECs were seeded on eight-well chamber slides (Falcon, Corning, New York) at the density of 20,000 cells per well and were grown to confluency. The next day, hPAECs were starved in a serum-reduced media (basal EBM-2 medium with 1% penicillin/streptomycin (P/S), Gibco, Thermo Fisher Scientific, Massachusetts) with 2% fetal bovine serum (FBS, Biowest, Nuaillé, France) for 2 h before the treatment. Cells were treated for 5, 30, and 90 min. Following treatment, cells were fixed in 4% (m/v) paraformaldehyde in PBS with Ca^2+^ and Mg^2+^ (Sigma-Aldrich, Missouri) for 25 min, were blocked with 5% (m/v) donkey serum and were permeabilized with 0.1% (m/v) Triton X in 3% (m/v) BSA in PBS at room temperature (RT). Chamber slides were incubated overnight with unconjugated primary antibodies at 4°C (VE-cadherin, 1:100, #AF938, R&D Systems, Minnesota; β1-integrin, 10 µg/mL, #MAB1997, clone MB1.2, Merck-Millipore, Massachusetts; and active β1-integrin, 10 µg/mL, #MAB2247, clone 12G10, Merck-Millipore, Massachusetts). Alexa Fluor 555 conjugated phalloidin (1:80; #A34055, Thermo Fisher Scientific, Massachusetts) was used to stain F-actin and was incubated together with secondary antibodies (all in 1:300, Thermo Fisher Scientific, Massachusetts) for 1 h in 0.1% Triton X (m/v) with 3% (m/v) BSA in PBS at RT. Chamber slides were counterstained and preserved using mounting media containing 4′,6-diamidino-2-phenylindole dihydrochloride (DAPI, Vectashield, Vector Laboratories, California).

hPAEC apoptosis was detected by incubating viable adherent ECs with CellEvent Caspase 3/7 detection reagent (1:700, #C10427, Thermo Fisher Scientific, Massachusetts), according to the manufacturer’s instructions. hPAECs were fixed as described above and counterstained and preserved using mounting media containing DAPI. All immunofluorescence images were acquired using A1 confocal laser microscope (Nikon, Tokyo, Japan) and visualized at ×60 magnification.

### FITC-Dextran Transwell Vascular Permeability Assay

hPAECs were seeded at the density of 20,000 cells per well into 96-well Transwell inserts (0.4 µm pore size, polycarbonate membrane, Corning, New York) precoated with 1% (m/v) gelatin. On the following day, hPAECs were put to serum-reduced media (basal EBM-2 medium with 1 P/S and 2% FBS) for 2 h, and stimulated with either vehicle or pentastatin for an additional 3 h at 37°C in a humidified incubator. Media was removed from the upper and lower compartments and washed twice with PBS containing Ca^2+^ and Mg^2+^ (Sigma-Aldrich, Missouri). FITC dextran permeability was assessed by adding 70 kDA fluorescein isothiocyanate-dextran (FITC-Dextran, 1 mg/mL, #46945, Sigma-Aldrich, Missouri) to the upper compartment. After 30 min of incubation at 37°C with FITC-Dextran, the fluorescence intensity in the lower compartment was measured at Ex/Em = 488/530 with a gain of 500 by using CLARIOstar Plus (BMG Labtech, Offenburg, Germany). Experiments were done in technical quadruplicates. Permeability is assessed by normalizing fluorescence intensity in arbitrary units to the vehicle, and data are represented as the “Permeability Index” in figure legends.

### RNA Isolation and qRT-PCR

Total RNA from hPAECs and hPASMCs were extracted using peqGOLD Total RNA Kit (Peqlab, Erlangen, Germany) or innuPREP RNA Mini Kit (IST Innuscreen, Berlin, Germany). The cDNA was reverse transcribed using a qScript cDNA Synthesis kit (QuantaBio, VWR, Vienna, Austria). Quantitative real-time polymerase chain reaction (qRT-PCR) was performed using the S’Green qPCR kit (Biozym, Hessisch Oldendorf, Germany) on LightCycler 480 (Roche Applied Science, Penzberg, Germany). The threshold cycle difference (ΔCT) was calculated on hPAECs material using the equation ΔCT_hPAECs_ = _Average_ [CT (β2M + PBGD) – CT Gene of Interest)] (in Fig. 2*A* and Supplemental Fig. S3*A*), on hPASMCs material using the formula ΔCT_hPASMCs_ = CT (β2M) – CT (Gene of Interest) (in Fig. 5*C* and Supplemental Fig. S6*C*), respectively. Beta-2-microglobulin (β2 M) and porphobilinogen deaminase (PBGD) were reference genes. The primer list is given in Table 2.

**Table 2.** Primer list

Gene	Forward Primer	Reverse Primer	Amplicon Length, bp
*ITGB1*	5′- CCGCGCGGAAAAGATGA-3′	5′- ACAATTTGGCCCTGCTTGTA-3′	149
*ITGB3*	5′- ATGGGACACAGCCAACAACC-3′	5′- ATTAAGTGCCCCGGTACGTG-3′	77
*ITGA1*	5′- TCCTCACTGTTGTTCTACGCTG-3′	5′- ACGGAGAACCAATAAGCACC-3′	150
*ITGA5*	5′- AAGGGAACCTCACTTACGGC-3′	5′- TAGGAGGCCATCTGTTCCCC-3′	91
*ITGAV*	5′- ATGCTCCATGTAGATCACAAGAT-3′	5′- AGCTACCAGGACCACCAAGA-3′	114
*JUNB*	5′- CCTCCCACTGGGGTCCAGGG-3′	5′- AGGTGGAAGGACTGGGCGCA-3′	111
*FOS*	5′- GGGATAGCCTCTCTTACTACCACT-3′	5′- GAAGTTGGCACTGGAGACGG-3′	110
*EGR1*	5′- ACCTGACCGCAGAGTCTTTT-3′	5′- GAGTGGTTTGGCTGGGGTAA-3′	84
*ZFP36*	5′- GACTGAGCTATGTCGGACCTT-3′	5′- GAGTTCCGTCTTGTATTTGGGG-3′	124
*CXCL1*	5′- CCAAACCGAAGTCATAGCCAC-3′	5′- AACTATGGGGGATGCAGGAT -3′	70
*CXCL2*	5′- CGGCAGGGAAATGTATGTGTG-3′	5′- GCTCTAACACAGAGGGAAACACT-3′	217
*CXCL3*	5′- CGCCCAAACCGAAGTCATAG-3′	5′- GCTCCCCTTGTTCAGTATCTTTT-3′	109
*CCL2*	5′- ACTCTCGCCTCCAGCATGAA-3′	5′- TTGATTGCATCTGGCTGAGC-3′	101
*CSF2*	5′- ACCTGCCTACAGACCCGCCT-3′	5′- GAAGTTTCCGGGGTTGGAGGGC-3′	128
*NFKBIA*	5′- TCAACAGAGTTACCTACCAGGGC-3′	5′- TCCTCTGTGAACTCCGTGAACTCT-3′	175
*C2CD4B*	5′- ACTGAAGAAGAGAGAAAGGCGCA-3′	5′- AATTCGGGGATGCGATTCGG-3′	156
*ACTA2*	5′- GCCTTGGTGTGTGACAATGG-3′	5′- ACCATCACCCCCTGATGTCT-3′	116
*CNN1*	5′- CCCCACGACATTTTTGAGGC-3′	5′- CACTCCCACGTTCACCTTGT-3′	123
*COL1A1*	5′- ACATGTTCAGCTTTGTGGACC-3′	5′- TGTACGCAGGTGATTGGTGG-3′	127
*COL3A1*	5′- GGTGTCCCAGGGAAAGATGG-3′	5′- CTCTCTCACCAGGGCTACCA-3′	145
*β2M*	5′- CCTGGAGGCTATCCAGCGTACTCC-3′	5′- TGTCGGATGGATGAAACCCAGACA-3′	113
*PBGD*	5′- CTGCAACGGCGAAGAAAA-3′	5′- AATCTTGTCCCCTGTGGTGG-3′	167

Bp, base pairs.

### Immunoprecipitation

Confluent, adherent hPAECs in full endothelial cell media were exposed to recombinant C-terminal NC1 fragment of collagen IVα5 polypeptide (NC1, #RPC141Hu01, Cloud-Clone, Wuhan, China) for 10 min, followed by crosslinking with 1% (m/v) PFA and quenching with 100 mM glycine for 50 min. Precleared protein lysates were incubated with 10 µg/mL monoclonal C-terminal collagen IVα5 antibody (#MAC141Hu21, Cloud-Clone, Wuhan, China). Protein complexes were captured with Protein G Sepharose 4 Fast Flow (GE Healthcare, Illinois). Pull-down proteins were blotted with an anti-β1-integrin subunit antibody (#MAB1997, clone MB1.2, Merck-Millipore, Massachusetts).

### β1-Integrin Subunit Solid-Phase Binding Assay

A microtiter plate was coated with 50 μL of 10 µg/mL NC1, pentastatin, random peptide control, or BSA in 50 mm NaHCO_3_, pH 9.6 at 4°C overnight. The plate was extensively washed with Tris-buffered saline (TBS; 25 mm Tris-Cl, 150 mm NaCl, pH 7.5), and nonspecific binding sites were blocked with 3% (m/v) BSA in TBS at RT for 1 h. α1β1-integrin (1 µg/mL; # CC1012, Chemicon, Sigma-Aldrich, Missouri) in TBS containing 1% (m/v) BSA, 1 mM MgCl_2_ and 2 mM CaCl_2_ was added to the wells, and β1-integrin subunit was allowed to bind to reagents immobilized on the microtiter plate at RT for 3 h. After extensive washing with TBS, bound β1-integrin subunit was detected using a biotinylated anti-β1-integrin subunit antibody [1:250 in TBS containing 1% (m/v) BSA, #BAF1778, R&D Systems, Minnesota] followed by peroxidase-labeled streptavidin [1:500 in TBS containing 1% (m/v) BSA, #P0397, DAKO, Agilent Technologies, California]. Final detection was performed using a 3,3′,5,5′-tetramethylbenzidine (TMB) substrate kit (Pierce, Thermo Fisher Scientific, Massachusetts), according to the manufacturer's instructions. The binding of β1-integrin subunit to BSA-coated wells was employed as a background control, against which values were normalized. Data were obtained from four independent experiments, which were performed in quadruplicates.

### Flow Cytometric β1-Integrin Subunit Staining

To determine the total and active β1-integrin subunit levels on the cell surface of hPAECs upon pentastatin treatment, hPAECs were detached and stimulated with random peptide control (50 µg/mL) for 30 min at 37°C in serum-reduced media (basal EBM-2 medium with 1% P/S, and 2% FBS). Then immediately, hPAECs were fixed with 4% (m/v) PFA on ice for 15 min. Subsequently, cells were divided into two equal parts and stained for 30 min with antibodies against total anti-β1-integrin subunit (10 µg/mL, #MAB1997, clone MB1.2, Merck-Millipore, Massachusetts) and against active anti-β1-integrin subunit (1:25, #MAB2247, clone 12G10, Merck-Millipore, Massachusetts). Donkey anti-rat and donkey anti-mouse Alexa 488 (1:300, Thermo Fisher Scientific, Massachusetts) were secondary antibodies. Fluorescence intensity was acquired on the BD Canto II (BD Biosciences, New Jersey).

### Cell Adhesion Assay

Adhesion assay with hPAECs was performed as previously described (1). In brief, hPAECs were allowed to adhere on 96-well plates at the density of 10,000 cells per well in full endothelial cell media containing vehicle or pentastatin (50 µg/mL) in a humidified incubator at 37°C for 30 min. After the removal of nonadherent cells, adherent hPAECs were fixed with 4% (m/v) formalin for 30 min on ice and consequently stained with 0.5% (m/v) crystal violet (Sigma-Aldrich, Missouri) in 25% (m/v) methanol for 20 min at RT. Experiments were performed in technical quadruplicates. Five images per well were taken at ×10 magnification. A blinded cell quantification was done using ImageJ (v1.53d, National Institutes of Health, Maryland).

### Flow Cytometric Assessment of Apoptosis

Cell survival of hPAECs via flow cytometry was assessed using two complementary methods: *1*) by using the CellEvent Caspase-3/7 green detection reagent to measure active caspase-3/7 and *2*) by using Annexin V-APC (AnxV, #A35110, Thermo Fisher Scientific, Massachusetts)/propidium iodide (PI, #130-093-233, Miltenyi Biotec, Bergisch Gladbach, Germany) double staining as previously described (21). hPAECs were put into serum-reduced media (basal EBM-2 medium with 1% P/S and 0.5% FBS) for 2 h before treatment. Treated-hPAECs cells were detached with trypsin and stained with Caspase 3/7 green detection reagent (1:700), Annexin V (1:25), and PI (1:100) in binding buffer (10 mM HEPES pH 7.4, 2.5 mM CaCl_2_, and 140 mM NaCl) according to the manufacturer’s instructions at the given time points. Data were acquired using the Cytoflex S (Beckmann Coulter, California) and BD FACS Canto II (BD Biosciences, New Jersey). Data analysis was performed using FlowJo (version 10.7, BD Bioscience, New Jersey).

### Thymidine Incorporation Assay

hPAEC proliferation was analyzed with a thymidine incorporation assay. hPAECs were seeded into 96-well plates at a density of 10,000 cells per well. The following day, cells were starved in serum-reduced media (basal EBM-2 medium with 1% P/S and 2% FBS for 30 min and treated with increasing concentrations of PS in starvation media with 1 µCi/mL ^3^H Thymidine (Biotrend Chemikalien, Cologne, Germany). After 24 h of incubation at 37°C, hPAECs were harvested as previously described (20). Radioactivity was determined in counts per minute (CPM) with a beta scintillation counter (Wallac 1450 MicroBeta TriLux, PerkinElmer, Massachusetts). Experiments were performed in technical quadruplicates.

### CCK-8 Proliferation Assay

hPASMCs proliferation was analyzed using Cell Counting Kit-8 (CCK-8, #CK04, Dojindo Molecular Technologies, Maryland, USA). hPASMCs were seeded into 96-well plates at a density of 5,000 cells per well. The following day, cells were starved in hPASMCs serum-deprived media [VascuLife Basal Medium (Lifeline Cell Technology, California) with 1% P/S] for 24 h. Subsequently, hPASMCs were treated with vehicle, PS, or RP for another 24 h and with supernatant of pentastatin-activated hPAECs for up to 48 h. At the treatment end point, 10 μL of CCK-8 was added to each well; cells were continuously incubated for 2 h more in the incubator. Afterward, the absorbance at 450 nm was determined using CLARIOstar Plus (BMG Labtech, Offenburg, Germany), according to the manufacturer’s instructions. Changes in proliferation were presented as “Proliferation Rate,” in which absorbance values are normalized to the vehicle-treated hPASMCs. Experiments were performed in technical quadruplicates.

### Bulk RNA Sequencing

Bulk-RNA sequencing on total RNA from hPAECs treated with vehicle/pentastatin (50 µg/mL) (*n* = 3/group) was performed on Illumina sequencing HiSeq 3000/HiSeq 4000 system at the CeMM Biomedical Sequencing Facility (Austrian Academy of Sciences-OeAW, Vienna, Austria). Differentially expressed genes (DEG) analysis was performed using the “DESeq2” package” (22). Enrichment analysis was performed using Enrichr version 3.0 (23–25), using the GO_Biological_Process as a reference data set. Significance was computed using Fisher’s exact test and Benjamini–Hochberg corrections, inferring an adjusted *P* value threshold 0.05. KEGG pathway analysis was computed using the R package “ClusterProfiler” version 4.0 (26), using single genes’ log2 fold-change values from the DEG analysis.

### Human Public Data Set Analysis

Single-cell RNA-sequencing (scRNA-seq) data set was obtained from PAs of patients with end-stage PAH and healthy controls (27), which can be found on GEO under the accession number GSE210248.

### Ex Vivo Isolated-Perfused and Ventilated Mouse Lung Model

Ex vivo experiments on isolated-perfused and ventilated mouse lungs from 9- to 12-wk-old male wild-type C57BL/6J (Charles River, Massachusetts) were performed, as previously described (28). Animal care and ex vivo experimental procedures on mouse lungs met EU guidelines (2010/63/EU) and local regulations; and were approved by the local authorities (Austrian Ministry of Education, Science, and Research, Vienna, Austria). In brief, animals were anesthetized with isoflurane (Piramal Critical Care, Bethlehem, PA) and killed by an overdose of intra-peritoneal injection of ketamine (200 mg/kg, Ketasol, LIVISTO, Senden, Germany) and xylazine (20 mg/kg, Rompun, Bayer, Leverkusen, Germany). The heart and lung were removed under artificial ventilation from exsanguinated animals; and were mounted in IPL-1 Core Isolated Perfused Lung System for Mouse (HSE Harvard Apparatus, Hugo Sacs Elektronik, March, Germany). Consequently, the lungs were perfused with sterile-warm (37°C) Krebs–Henseleit–hydroxyethyl amylopectin buffer (28), and the left arterial pressure was set to +2.2 cmH_2_O. Perfused lungs were ventilated by negative pressure (−4.5 to −9.0 cmH_2_O) in a closed chamber. Lung volume was controlled with a ∼9 mL/kg tidal volume, an end-expiratory pressure of −2 cmH_2_O, a respiratory rate of 90 breaths/min, and hyperinflation (−24 cmH_2_O, 4-min interval). After an initial steady-state period of 20 min of one-way perfusion, the system was switched to recirculation for 10 min (1 mL/min) before the addition of either vehicle (DMSO), PS (50 µg/mL) or RP (50 µg/mL) into the circulation. Compounds were recirculated in the system for a maximum of 120 min at 37°C. Lung function parameters, including compliance, tidal volume (TV), and mean pulmonary arterial pressure (mPAP), were monitored throughout the experiment by using PULMODYN software (HSE Harvard Apparatus, Hugo Sachs Elektronik, March, Germany). To evaluate the vascular permeability, Evans blue (0.675 mg/mL, Sigma-Aldrich, Missouri) was recirculated for 15 min at the end of the experiment (29). The right lung of the vehicle-pentastatin-perfused lungs was lavaged with 500 µL PBS containing protease and phosphatase inhibitors, and bronchoalveolar lavage fluid (BALF) was collected. Evans Blue levels in BALF were measured at absorbance 620 nm using CLARIOstar Plus (BMG Labtech, Cologne, Germany). Total protein concentration in the circulation buffer was measured using a BCA protein assay kit (#71825, Merck-Millipore, Massachusetts).

### Transmission Electron Microscopy

Isolated-perfused mouse lung samples were fixed with 2% (m/v) paraformaldehyde and 2.5% (m/v) glutaraldehyde in 0.1 M cacodylate buffer, pH 7.2, and post-fixed in osmium tetroxide. Consequently, samples were dehydrated, embedded in epoxy resin, and cut into 70 nm ultrathin sections (UC 7Ultramicrotome, Leica Microsystems, Wetzlar, Germany). Following uranyl acetate staining, images were taken using transmission electron microscopy (TEM) (EM 900 or EM109, Zeiss, Jena, Germany).

### Immunofluorescence Staining on Mouse Lungs

Formalin-fixed paraffin-embedded isolated-perfused mouse lung tissues were cut into 2.5-µm thick serial sections. Sections were deparaffinized in RotiClear (Carl Roth, Karlsruhe, Germany) and rehydrated in decreasing ethanol concentration. For immunofluorescence staining, tissue sections were subjected to heat-induced antigen retrieval (DAKO pH 6.0, Agilent Technologies, Agilent Technologies, California) at 95°C for 20 min, followed by endogenous enzyme blocking (BLOXALL, Vector Laboratories, California) for 20 min at RT, and blocking-permeabilization with 0.1% (m/v) Triton X in 3% (m/v) BSA in PBS for 1 h at RT. Primary antibodies for VE-cadherin (1:100, AF1002, R&D Systems, Minnesota), vWF-555 (1:100, clone F8/86, M0616, DAKO, Agilent Technologies, California), and cleaved-caspase 3 (1:200, #9661, Cell Signaling Technology, Massachusetts) were diluted in 3% (m/v) BSA in PBS. They were applied overnight at 4°C followed by secondary antibody (1:300, all from Thermo Fisher Scientific, Massachusetts) incubation for 1 h at RT the next day. DAPI (Vectashiled, Vector Laboratories, California) was used as a counterstain. Images were obtained by using Nikon A1 confocal microscope (Nikon, Tokyo, Japan).

### Immunohistochemistry on the Human Lung Tissue

Formalin-fixed paraffin-embedded human lung tissues were cut into 2.5-µm thick serial sections. A standard immunohistochemistry protocol was applied. Sections were deparaffinized in RotiClear (Carl Roth, Karlsruhe, Germany). Deparaffinization was followed by decreasing ethanol rehydration and antigen retrieval (DAKO, Agilent Technologies, California). Endogenous peroxidase activity was blocked using BLOXALL (Vector Laboratories, California) for 20 min at RT. Consequently, tissue blocking was performed for 1 h at RT using 2.5% (m/v) horse serum (Vector Laboratories, California). Tissues were incubated with unconjugated primary antibodies overnight at 4°C, followed by secondary antibody incubation for 1 h at RT the next day. The signal was developed using Vector NovaRED, ImmPACTVIP, and ImmPACTDAB (all from Vector Laboratories, California). Mayer’s hematoxylin solution (Sigma-Aldrich, Missouri) or methyl green (Sigma-Aldrich, Missouri) were used as counterstain. Slides were scanned using a slide scanning microscope (BX61VS and BX-UCB, Olympus Life Science, Tokyo, Japan) and visualized at ×40 magnification. Detailed information on antibodies, antigen retrieval, and signal-developing reagents can be found in Table 3.

**Table 3.** Details of immunohistochemistry

Antibody	Company	Catalog No.	AR	Host	Dilution	Detection
Type IV Collagen	MyBioSource	#MBS534518	DAKO pH 6.0	Rb IgG	1:500	Vector NovaRed
NC1-CollagenIVα5	CloudClone	#MAC141Hu21	DAKO pH 9.0	Ms IgG1κ	1:100	Vector NovaRed
αSMA	Fisher Scientific	#RB-9010-P1	Pepsin	Rb IgG	1:100	ImmPACTVIP
VWF (Clone F8/86)	DAKO	#M0616	Pepsin	Ms IgG1	1:1,000	ImmPACTDAB

AR, antigen retrieval; NC1-CollagenIVα5, C-terminal end of CollagenIVα5; αSMA, alpha-smooth muscle actin; VWF, von Willebrand factor.

### Measurements of ColIVα5 and NC1-ColIVα5 Polypeptides in Plasma

To avoid proteolytic cleavage and degradation of ColIVα5 polypeptide and NC1-ColIVα5, plasma from sex-age-matched non-PH (controls) and outpatients with IPAH were collected in a unique blood collection system containing a cocktail of proteases, esterases, and DPP-IV inhibitors (BD P800 Blood Collection System, #366420, BD Biosciences, California). A commercial ELISA kit (#SEC141Hu, Cloud-Clone, Wuhan, China) was used to measure ColIVα5 and NC1-ColIVα5 levels in the plasma of controls (*n* = 10) and outpatients with IPAH (*n* = 10). Samples were diluted at 1:80 and measured according to the manufacturer’s instructions. A detailed description of the patient’s characteristics used for ELISA can be found in Table 4.

**Table 4.** Patient characteristics of controls and outpatients with IPAH for ELISA

	Control, *n* = 10	IPAH, *n* = 10
Age	53.0 [41.0–57.0] (10)	59.0 [51.3–68.0] (10)
Sex, F/M	8 (80 %)/2 (20 %)	10 (100.0 %)/0 (0.0 %)
Body height, cm	170.0 [165.0–172.0] (5)	164.2 [161.0–167.8] (10)
Body weight, kg	70.0 [65.0–72.0] (5)	81.5 [67.5–99.5] (10)
BMI, kg/m^2^	25.2 [24.3–26.6] (5)	30.3 [23.4–36.0] (10)
mPAP, mmHg	N/A	39.6 [25.5–51.5] (10)
PAWP, mmHg	N/A	9.0 [7.3–10.8] (10)
PVR, wood units	N/A	7.2 [2.9–9.5] (10)
CO, L/min	N/A	4.65 [3.58–5.54] (10)
CI, 1 min^−1^·m^−2^	N/A	2.7 [2.4–3.2] (10)
Po_2,_ mmHg	N/A	62.3 [55.8–65.1] (10)
Pco_2,_ mmHg	N/A	33.9 [31.8–36.3] (10)
6MWD, m	N/A	355.50 [212.25–501] (8)
NT-proBNP, pg/mL	N/A	707.9 [161.8–510.0] (10)
PAH treatment	N/A	6 (60 %)

Unless otherwise stated, data are presented as *n* (%), median [IQR], and (*n*) to represent the number of individuals that data was obtained from within the group. IPAH, idiopathic pulmonary arterial hypertension; F/M, female/male; BMI, body mass index; N/A, data is not available for the entry; mPAP, mean pulmonary arterial pressure; mmHg, millimeter of mercury; PAWP, pulmonary arterial wedge pressure; PVR, pulmonary vascular resistance; CO, cardiac output; CI, cardiac index; Po_2_, partial pressure of oxygen; Pco_2_, partial pressure of carbon dioxide; 6MWD, six-minute walk test; NT-proBNP, B-type natriuretic peptide; PAH, pulmonary arterial hypertension.

### Statistical Analysis

Data were analyzed at least from three independent experiments per assay. Variables with assumed approximate log-normal distribution, such as concentrations and intensities, as well as ratios of such variables, have been log-transformed before testing hypotheses about mean differences. Residuals from each analysis were tested for normality using a Shapiro–Wilks test (α = 0.05) and D’Agostino’s K-squared test (α = 0.05). The reasonability of the Gauss–Markov assumptions has been checked with residual diagnostic plots. The parametric statistics was applied on normally distributed samples using a *t* test and one-way/two-way ANOVA following post hoc tests to perform multiple comparisons using Prism 8.0 (GraphPad Software). Specific tests used for each data set are indicated in the figure legend. Probability values of *P* < 0.05 were considered statistically significant.

## RESULTS

### C-terminal NC1 Fragment of ColIVα5 and Its Bioactive Peptide Pentastatin Impair Human Pulmonary Arterial Endothelial Barrier Integrity

**Figure 1.**
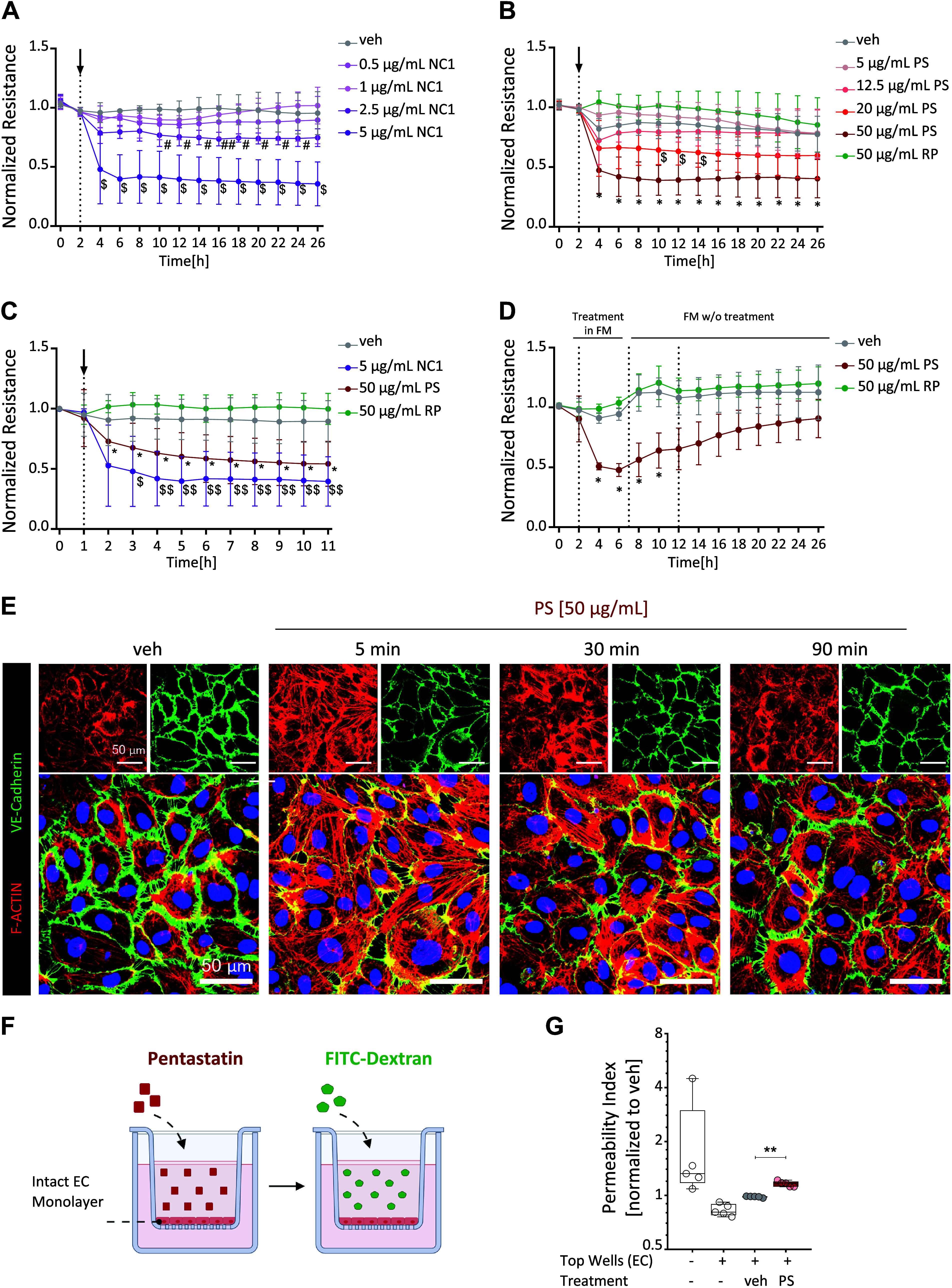
Pentastatin (PS) reduces human pulmonary arterial endothelial cell (hPAEC) barrier integrity in vitro. *A*: normalized endothelial resistance following treatment with NC1-ColIVα5 fragment (NC1) and vehicle (veh, PBS). veh vs. 2.5 µg/mL NC1: #*P* < 0.05; veh vs. 5 µg/mL NC1: $*P* < 0.05 or *P* < 0.01; determined by two-way ANOVA followed by Dunnett’s post hoc test; *n* = 4–6 independent experiments from *n* = 5 different hPAECs. *B*: normalized endothelial resistance following treatment with vehicle (veh, DMSO), PS, or random peptide (RP). veh vs. 20 µg/mL PS: §*P* < 0.05; veh vs. 50 µg/mL PS: *P < 0.05, **P < 0.01 or *P* < 0.001; determined by two-way ANOVA followed by Dunnett’s post hoc test; *n* = 5 independent experiments from *n* = 5 different hPAECs. *C*: normalized endothelial resistance following treatment with veh, NC1 (5 µg/mL), PS (50 µg/mL) or with RP (50 µg/mL). veh vs. 5 µg/mL NC1: $*P* < 0.05, $$*P* < 0.01; determined by two-way ANOVA followed by Tukey’s post hoc test; *n* = 11 independent experiments from *n* = 8 different hPAECs for PS, *n* = 4–6 independent experiments from *n* = 5 different hPAECs for others. *D*: monitoring 5 h treatment with veh, PS (50 µg/mL) or with RP (50 µg/mL) followed by 20 h of recovery phase in full media without a treatment. veh vs. 50 µg/mL PS: *P < 0.05 or *P* < 0.01; determined by two-way ANOVA followed by Tukey’s post hoc test; *n* = 7 independent experiments from *n* = 3 different hPAECs. Normalized hPAEC resistance measured in real-time using the electrical cell-substrate impedance sensing (ECIS) system. When a stable endothelial resistance is established, a baseline endothelial resistance was measured 2 h before treatment, and endothelial resistance at any given time point was normalized to baseline. Error bars represent standard deviation (SD). *E*: visualization of adherens junctions (VE-cadherin, in green) and stress fibers (F-actin, phalloidin, in red) in hPAECs following veh or PS (50 µg/mL) treatment. Images are representative of *n* = 3 independent experiments from *n* = 3 different hPAECs. Scale bars: 50 µm. *F*: schematic representation of experimental setup for FITC-Dextran assay. Created with BioRender and published with permission. *G*: FITC-Dextran (70 kDa) was measured in fluorescence arbitrary units and normalized to veh. *Y*-axis set to log2 scale. veh vs. PS (50 µg/mL): *P < 0.05; determined by one-way ANOVA for repeated measures with Tukey’s post hoc test; *n* = 5 independent experiments from *n* = 3 different hPAECs. DMSO, dimethyl sulfoxide; EC, endothelial cell.

Endothelial dysfunction due to impaired endothelial barrier integrity is increasingly associated with the pathophysiology of pulmonary vascular diseases, such as PH (30–33). Therefore, this study investigated whether treatment with the NC1-ColIVα5 fragment and its shorter pentastatin peptide (sequence displayed in Supplemental Fig. S1) directly affects endothelial cell monolayer integrity by monitoring real-time endothelial cell resistance using the ECIS system (Fig. 1, *A*–*D*). The applied synthetic peptides were pre-tested for endotoxin levels (<1EU/µg, as shown in Supplemental Fig. S2, *A* and *B*).

In a concentration range between 0.5 and 5 µg/mL (the equivalent of 20–200 nM), the effect of the NC1-ColIVα5 fragment on hPAEC barrier integrity was tested (Fig. 1*A*). The highest concentration gave a strong and immediate barrier disruption as early as 30 min. Similarly, the shorter bioactive pentastatin peptide exerted an immediate and long-lasting barrier-disrupting effect in a concentration-dependent manner (Fig. 1*B*). The most pronounced effect of pentastatin was observed at 50 µg/mL (equivalent to 20 µM). The random peptide control (RP, 50 µg/mL) did not alter endothelial cell resistance at equivalent concentrations to pentastatin (Fig. 1, *B*–*D*). Together, this suggests that NC1-ColIVα5 fragments mediate endothelial barrier integrity via the bioactive 20 amino acids long pentastatin sequence.

To determine if the effect of pentastatin on endothelial monolayer can be reversed, recovery experiments were performed. hPAECs were exposed to pentastatin for 5 h, resulting in a steady decrease in endothelial cell resistance. Subsequently, pentastatin was removed, the media was replenished, and endothelial cell resistance was monitored for the following 24 h. Supplementation with fresh full media improved endothelial cell resistance in all investigated conditions, including pentastatin-treated endothelial cells (Fig. 1*D*).

The endothelial barrier is tightly regulated by adherens junctions, namely VE-cadherin, and the contractile cytoskeleton. Therefore, the effect of pentastatin on adherens junctions and the cytoskeleton was investigated using immunofluorescence staining of VE-cadherin and F-actin. The stimulation of hPAEC with pentastatin resulted in the opening of cell junctions while simultaneously inducing stress fiber formation (Fig. 1*E*). The effect was most pronounced in the early response (Fig. 1*E*, 5 min, compared with 30 and 90 min of treatment).

Loss of endothelial cell integrity via dysregulation of adherens junctions and actin cytoskeleton may lead to paracellular permeability, allowing passage of big molecules and immune cells from the plasma to the tissue through the endothelium (34). Therefore, to identify whether the changes in trans-endothelial resistance would also allow a passage for macromolecules, a proof-of-concept study was performed evaluating the passage of 70 kDa dextran through an intact endothelial cell monolayer following pentastatin treatment (Fig. 1*F*). Indeed, 3 h of pentastatin treatment increased the passage of dextran molecules through the endothelial cell layer, indicating pentastatin-exposure enhances paracellular permeability in hPAEC (Fig. 1*G*).

### Pentastatin Action Involves the Activation of β1-Integrin Subunit

**Figure 2.**
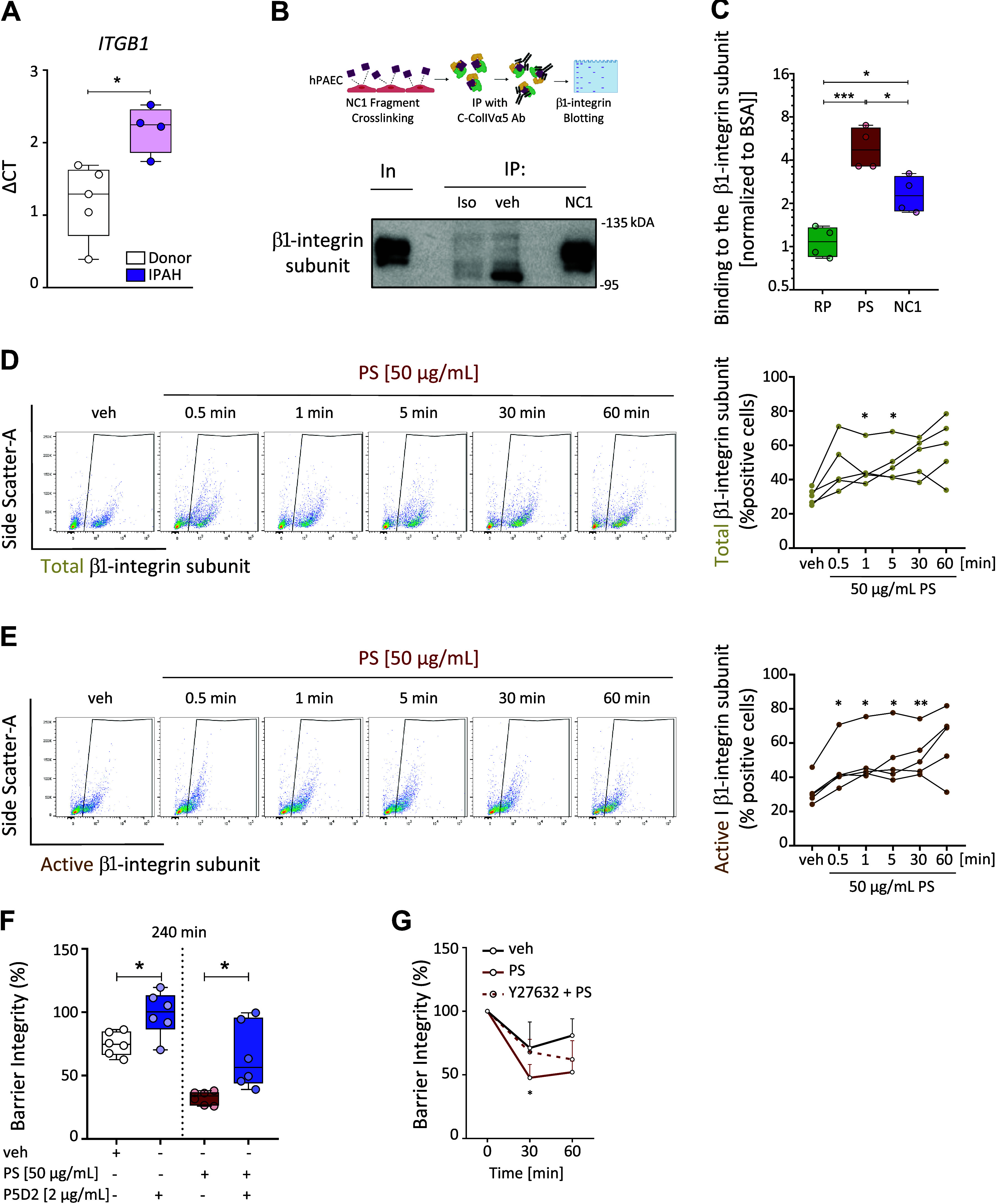
Pentastatin (PS) promotes β1-integrin subunit activation on human pulmonary arterial endothelial cells (hPAECs). *A*: *ITGB1* gene expression in healthy-hPAECs (*n* = 5) compared with IPAH-hPAECs (*n* = 4 derived from *n* = 3 different IPAH-hPAECs. *P < 0.05; determined by unpaired *t* test. IPAH, idiopathic pulmonary arterial hypertension; *ITGB1*, integrin subunit beta 1. *B*: pull-down of NC1 with β1-integrin subunit along with schematic representation of immunoprecipitation workflow. hPAECs were exposed to either vehicle (veh) or NC1 for 10 min. Consequently, hPAECs/NC1 were cross-linked and protein complexes were immunoprecipitated. IP, immunoprecipitated material; Iso, isotype-matched control. Created with BioRender and published with permission. *C*: direct-binding of β1-integrin subunit to pentastatin (PS) and to NC1 was revealed using a solid-phase binding assay. β1-integrin subunit was allowed to bind surface coated with equal concentration of veh, NC1, PS, random peptide (RP), and bovine serum albumin (BSA). Binding was measured in absorbance, normalized to BSA. *Y*-axis set to log2 scale. *P < 0.05, ***P < 0.001; determined by one-way ANOVA with Tukey’s post hoc test. *D* and *E*: hPAECs were exposed to veh or PS (50 µg/mL), and β1-integrin subunit levels on the cell surface were analyzed by flow cytometry. Representative dot blot plot from a single experiment and quantification of total β1-integrin subunit (clone MB1.2; *D*) and active β1-integrin subunit (clone 12G10; *E*) on cell surface upon PS stimulation. veh vs. PS: *P < 0.05 or **P < 0.01; determined by one-way ANOVA for repeated measured followed by Tukey’s post hoc; *n* = 5 independent experiments from *n* = 3 different hPAECs. *F*: barrier integrity of hPAECs pretreated with β1-integrin neutralizing antibody (2 µg/mL, clone P5D2) for 3 h prior to veh or PS (50 µg/mL) treatment at 240 min after veh/PS the stimulation. Barrier integrity was calculated by percentage (%) of the normalized endothelial resistance at given time point compared with baseline. *P < 0.05; determined by paired *t* test; *n* = 6 independent experiments from *n* = 5 different hPAECs. *G*: barrier integrity of hPAECs pretreated with ROCK inhibitor Y-27632 (20 µM) for about 70 min before veh or PS (50 µg/mL) at 30 and 60 min after the veh/PS stimulation presented as %. PS effect alone vs. PS effect with Y-27632 on hPAEC monolayer: *P < 0.05; determined by two-way ANOVA followed by Tukey’s post hoc test; *n* = 6 independent experiments from *n* = 3 different hPAECs donors. Error bars represent the standard deviation (SD).

Integrins, heterodimer transmembrane receptors containing α and β chains, are indispensable in manifesting cell adhesion via transferring cues between cells or cells and their surrounding matrix. In the endothelium, integrin signaling has been linked to VE-cadherin organization and endothelial barrier integrity (35–37). Therefore, the gene expression of several integrin subunits was analyzed in isolated hPAECs from healthy controls and patients with IPAH. Consequently, gene expression of β1- (encoded by *ITGB1*) and α_1_- (encoded by *ITGA1*) integrin subunits were found to be higher in hPAECs isolated from patients with IPAH compared with healthy controls (Fig. 2*A*; Supplemental Fig. S3*A*). Notably, the α1-integrin subunit is known to dimerize with the β1-integrin subunit, the major β-integrin subunit on endothelial cells. As these findings pinpoint toward a major role for the β1-integrin subunit in IPAH, receptor-ligand interactions of the β1-integrin subunit and NC1-ColIVα5/pentastatin were investigated in hPAECs.

Here, hPAECs were cross-linked with the commercially available recombinant NC1 fragment of ColIVα5 and immunoprecipitated with a ColIVα5 antibody, which was raised against the NC1 domain of ColIVα5 (Gly1461 ∼ Thr1685) (Fig. 2*B*). The β1-integrin subunit was detected in the immunoprecipitated complex, suggesting an interaction between the β1-integrin subunit and NC1-ColIVα5/pentastatin on hPAECs (Fig. 2*B*). Furthermore, a direct interaction between the β1-integrin subunit and pentastatin was identified by performing a solid-phase binding assay, where the random peptide control did not show any binding affinity to the β1-integrin subunit (Fig. 2*C*). Together, these results indicate that the β1-integrin subunit is a receptor for NC1-ColIVα5/pentastatin in hPAECs.

To further delineate whether the binding of NC1-ColIVα5/pentastatin would also result in the functional activation of β1-integrins, its dynamics were investigated in hPAECs. Indeed, treatment with pentastatin increased the cell surface levels of both the total and active β1-integrin subunit (extended-open conformation) in a time-dependent manner (Fig. 2, *D* and *E*), as determined by flow cytometry. Microscopical visualization on endothelial monolayers revealed a cellular redistribution of the total β1-integrin subunit from spot-like to rather elongated patches following 90 min of pentastatin treatment (Supplemental Fig. S3*B*, *top*). Similarly, after pentastatin treatment, a stronger active β1-integrin subunit signal (at 90 min) was located at the site of focal adhesions, indicating integrin clustering. Alongside, considerable levels of the active β1-integrin subunit were found in the cytoplasm, yet distributed, which may pinpoint toward internalization (Supplemental Fig. S3*B*, *bottom*). In line, functional blocking of the β1-integrin subunit activation site using the neutralizing antibody P5D2 revealed a partial reduction in pentastatin-mediated barrier disruption (Fig. 2*F* and Supplemental Fig. S3*C*). These findings suggest that the binding of NC1-ColIVα5/pentastatin to the β1-integrin subunit is followed by β1-integrin subunit activation in hPAECs, thereby contributing to pentastatin-induced loss of hPAEC resistance.

### Pentastatin Partially Acts via the Engagement of Rho-ROCK-Myosin Light Chain Signaling

Actin contraction and increased endothelial cell permeability can be facilitated by β1-integrin subunit-mediated phosphorylation of the myosin light chain (MLC) (38, 39). Indeed, pentastatin treatment on hPAECs led to phosphorylation of MLC after 5 and 40 min (Supplemental Fig. S4, *A* and *B*). Therefore, to investigate whether the Rho/ROCK pathway was associated with pentastatin-induced decrease in endothelial resistance, hPAECs were pretreated with 20 µM Y-27632 (ROCK inhibitor; concentration was determined by inhibition of thrombin-induced barrier disruption as shown in Supplemental Fig. S5, *A* and *B*). Pretreatment with Y-27632 did not inhibit the rapid early drop in endothelial resistance mediated by pentastatin; however, it promoted quicker partial barrier recovery (Fig. 2*G* and Supplemental Fig. S5*C*), suggesting a partial involvement of the Rho/ROCK pathway in the long-term barrier disruption mediated by pentastatin.

### Pentastatin Leads to Early Endothelial Cell Activation In Vitro

**Figure 3.**
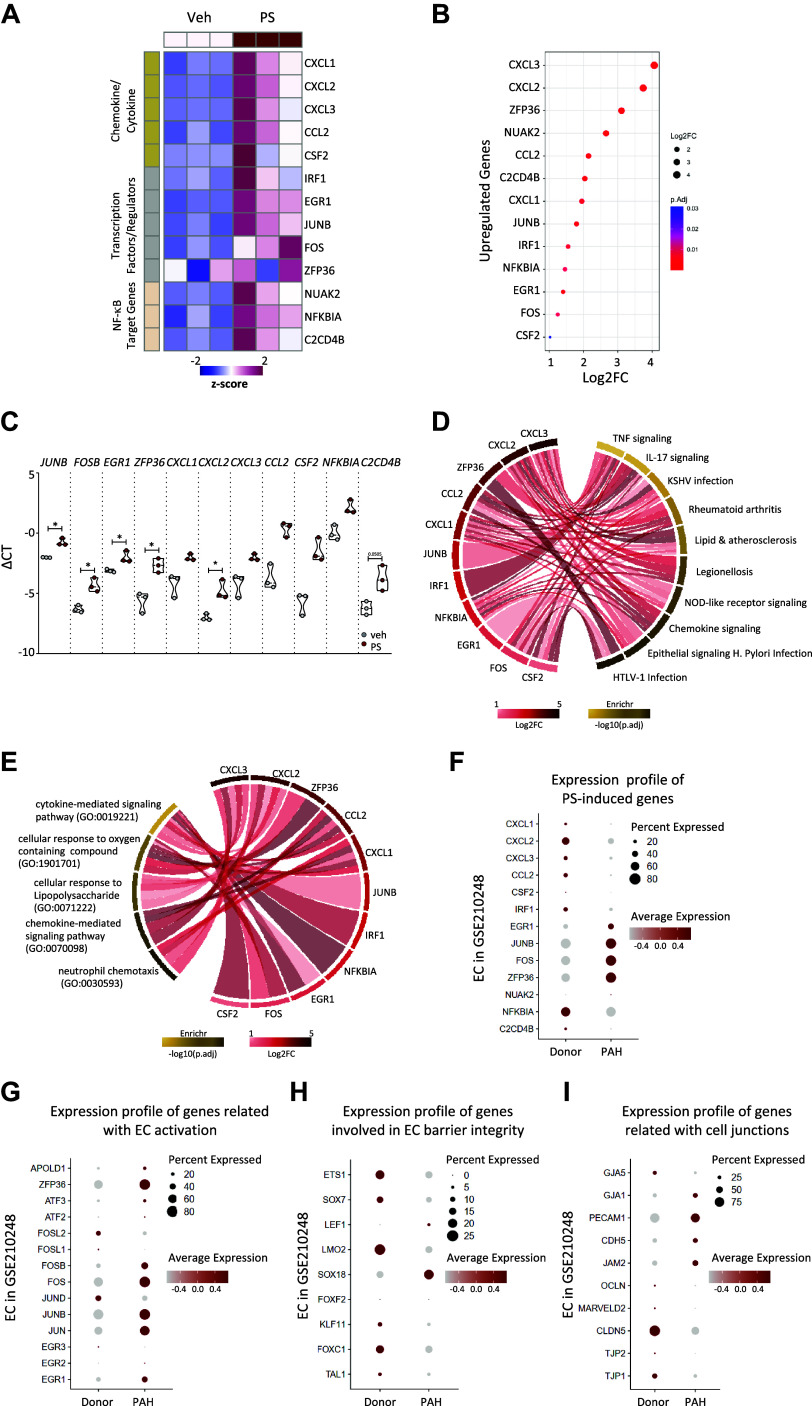
Pentastatin upregulates immediate early genes and cytokines in human pulmonary arterial endothelial cells (hPAECs). *A*: heatmap representing differentially expressed genes (DEGs) in hPAECs treated with vehicle (veh) or pentastatin (PS, 50 µg/mL) for 90 min, scaled by row. *B*: bubble plot representing log2 fold change of the DEGs. Significance threshold: *P.*adjust < 0.05. *C*: validation of pentastatin target genes by quantitative real-time PCR analysis, samples are same as in *A* and *B*. veh vs. PS: *P < 0.05, determined by paired *t* test. *D*: the KEGG pathway analysis. *E*: gene ontology (GO) enrichment analysis for biological processes illustrated using chord diagram. Analysis was performed in Enrichr with Fisher’s exact test with Benjamini–Hochberg multiple-testing correction; significance threshold: *P*.adjust < 0.05. Links between enriched KEGG pathways/biological processes and genes are color-coded according to Log2FC from DEseq2 as determined by the independent weighting hypothesis method. *F*–*I*: dot plots for the EC-compartment of end-stage pulmonary arteries (PAs) from patients with pulmonary arterial hypertension (PAH) compared with healthy donors. *F*: pentastatin target genes. *G*: 14 transcription factors (TFs) related to EC activation. *H*: 9 TFs related to EC barrier integrity. *I*: 10 genes encoding EC junction proteins. The dot size represents the percentage of cells expressing the gene; the color gradient represents the average expression across the data set. Data were recruited from a publicly available single-cell RNA sequencing data set [GSE210248, (27)].

Given these strong effects mediated by the pentastatin on hPAEC barrier function, an unbiased RNA sequencing approach was performed on hPAECs treated with vehicle or pentastatin for 90 min. Pentastatin upregulated the transcription of genes including chemokines and cytokines (*CXCL1-3*, *CCL2*, and *CSF2*), transcription factors related to endothelial cell activation, also known as immediate early genes (*EGR1-2*, *IRF1*, *JUNB*, and *FOS*), and genes associated with NF-κB, a cascade related to apoptosis regulation and pro-inflammatory responses (*NFKBIA*, *NUAK2*, *C2CD4B*) (Fig. 3, *A* and *B*). The upregulation of those genes was further validated using quantitative real-time PCR (Fig. 3*C*). KEGG pathway analysis further revealed a strong link to early immune activation and inflammatory response by activating pathways involved in cytokine signaling (such as TNF-α and IL-17 signaling), chemokine signaling, and bacterial infections (Fig. 3*D*). This was recapitulated by gene ontology (GO) enrichment analysis, revealing activation of biological processes related to the response to cytokines and chemokines, bacterial stimuli, lipids, as well as processes involved in granulocyte chemotaxis (Fig. 3*E*). Taken together, this strongly suggested that pentastatin may act as a rapid endothelial cell activator, upregulating early immediate genes and cytokines.

Having identified a gene signature of pentastatin, the question was whether the same genetic programs would be activated in endothelial cells of remodeled vessels from patients with PAH. For this purpose, we analyzed our recently published single-cell RNA data set obtained from PAs of patients with end-stage PAH and healthy controls [GSE210248, (27)]. This analysis revealed that some pentastatin target genes which are involved in early immediate gene response and endothelial cell activation, namely *JUNB*, *FOS*, *EGR1* and *ZFP36* (as identified in Fig. 3, *A*–*C*), were upregulated in the endothelial cell compartment of end-stage PAH-PAs (Fig. 3, *F* and *G*), while transcription factors that are known to be crucial for maintaining endothelial cell barrier integrity [*ETS1*, *SOX7*, *LEF1*, *LMO2*, *SOX18*, *FOXF2*, *KLF11*, *FOXC1*, *TAL1*, (40)] were downregulated (Fig. 3*H*). This was accompanied by a switch in the expression of genes encoding adhesion molecules, characterized by the downregulation of tight junction genes and upregulation of genes encoding adherens junction proteins (Fig. 3*I*).

### Pentastatin Induces Apoptosis in hPAECs

**Figure 4.**
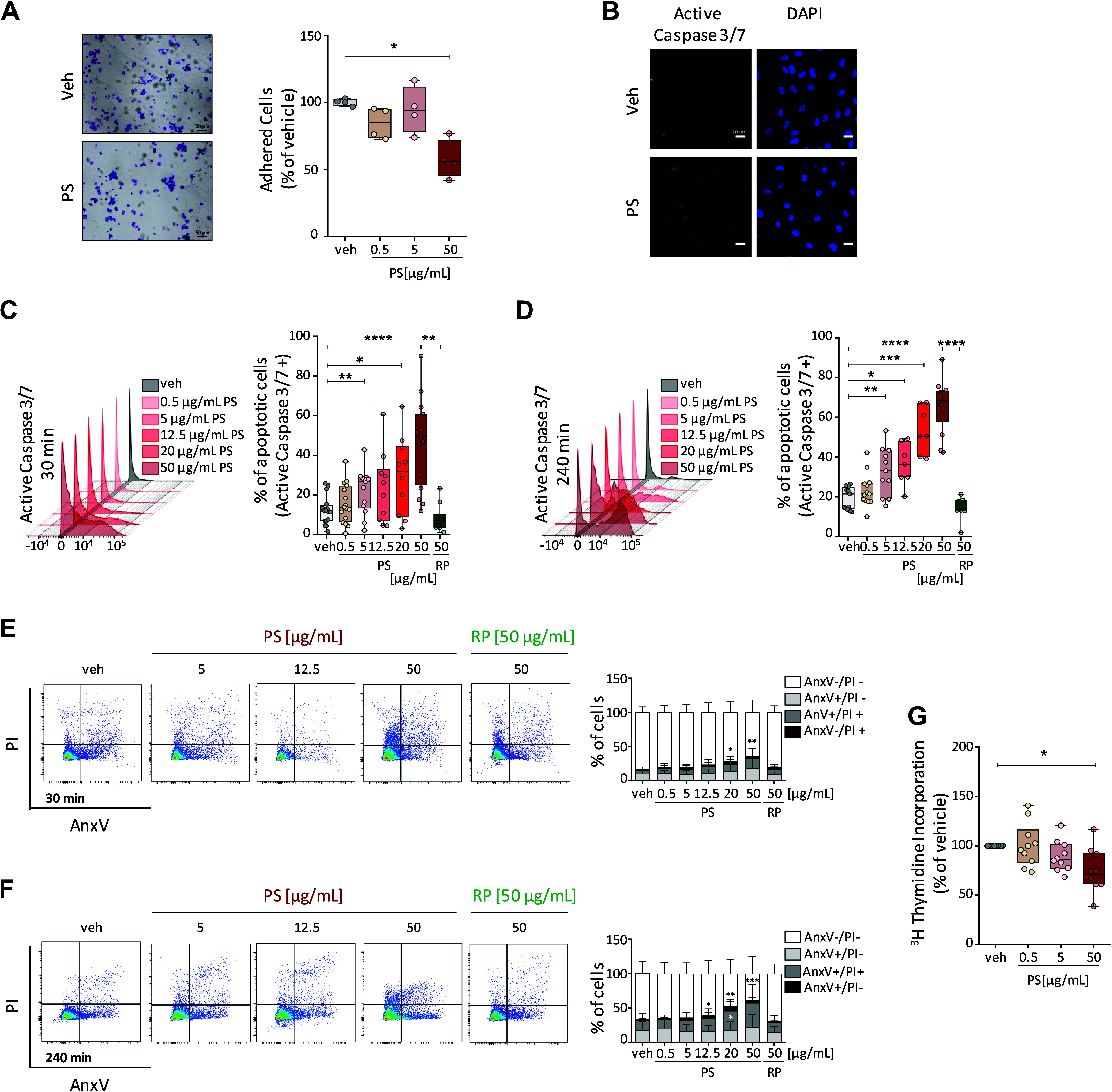
Pentastatin (PS) induces cell death in human pulmonary arterial endothelial cells (hPAECs). *A*: representative micrographs and quantification of hPAECs adhesion following treatment with veh or increasing concentration of PS (0.5–50 µg/mL) for 30 min. Scale bars: 50 µm. *P < 0.05; determined by one-way ANOVA for repeated measures followed by Tukey’s post hoc test; representative of *n* = 4 independent experiments from *n* = 4 different hPAECs. *B*: visualization of active caspase 3/7 in hPAECs after stimulation with either veh or PS (50 µg/mL) for 30 min. Scale bars: 20 µm. *C*–*F*: hPAECs were stimulated with veh or increasing concentration of PS (0.5–50 µg/mL) for 30 and 240 min. Active caspase 3/7 (*C* and *D*) and Annexin V/PI double staining (*E* and *F*) analyzed by flow cytometry. In *D–G*, *P < 0.05, **P < 0.01, ***P < 0.001, and ****P < 0.0001; determined by mixed-effect analysis of variance (split-plot ANOVA) with Dunnett’s post hoc test; in C and *D*, PS (50 µg/mL) vs. RP (50 µg/mL): **P < 0.01 and ****P < 0.0001; determined by paired *t* test; *n* = 6 independent experiments from *n* = 4 different hPAECs. Error bars represent standard deviation in *E* and *F*. *G*: proliferation of hPAECs were assessed by ^3^H-thymidine incorporation after 24 h stimulation of increasing concentration of PS (0.5–50 µg/mL). Thymidine counts were normalized to veh and presented as percentage (%). *P < 0.05; determined one-way ANOVA for repeated measures with Tukey’s post hoc test; *n* = 10 independent experiments from *n* = 7 different hPAECs. DAPI, 4′,6-diamidino-2-phenylindole dihydrochloride; AnxV/PI, Annexin V/propidium iodide.

Pentastatin treatment on hPAECs led to impaired cell adhesion (Fig. 4*A*) and the appearance of cells positive for active caspase 3/7 (Fig. 4*B*). As these results suggested an influence of pentastatin on cell viability, the concentration and time dependence of pentastatin on hPAEC apoptosis were next investigated using flow cytometry. After 30 and 240 min, a pro-apoptotic effect of pentastatin was observed in concentrations between 5 and 50 µg/mL (Fig. 4, *C* and *D*, bar graphs).

Annexin V/PI flow cytometry staining confirmed the induction of apoptosis (AnxV+), rather than necrosis (AnxV-/PI+), by pentastatin in hPAECs (Fig. 4, *E* and *F*). In contrast, the random peptide control did not affect cell viability (Fig. 4, *C*–*F*). In line with the pro-apoptotic effect of pentastatin, we observed decreased cell proliferation after 24 h of treatment with the highest pentastatin concentration (50 µg/mL) (Fig. 4*G*).

### Pentastatin Induces Proliferation and Gene Expression of Extracellular Matrix Genes in hPASMCs

**Figure 5.**
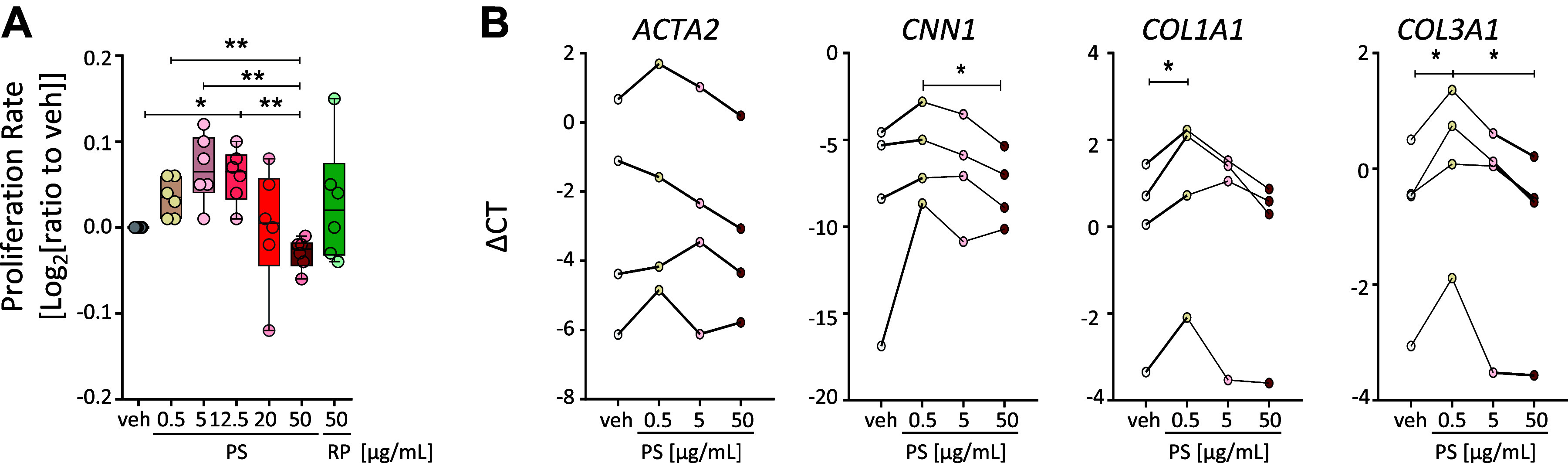
Pentastatin (PS) induces proliferation of human pulmonary smooth muscle cells (hPASMCs) and induces gene expression of fibrillar collagens. *A*: proliferation of hPASMCs were assessed by CCK-8 assay after 24 h stimulation with vehicle (veh), increasing concentration of pentastatin (PS, 0.5–50 µg/mL) or random peptide (RP) (50 µg/mL). Absorbance is normalized to vehicle and represented in log2. *P < 0.05, **P <0.01; determined by one-way ANOVA for repeated measures followed by Tukey’s post hoc test; *n* = 6 independent experiments from *n* = 4 different hPASMCs. *B*: quantitative real-time PCR analysis of *ACTA2, CNN1*, *COL1A1*, and *COL3A1* on hPASMCs after 24 h stimulation with either veh or increasing concentration of PS (0.5–50 µg/mL). *P < 0.05; determined by one-way ANOVA for repeated measures followed by Tukey’s post hoc analysis. *N* = 4 independent experiments from *n* = 4 different hPASMCs. *ACTA2*, actin alpha 2, smooth muscle; *CNN1*, calponin 1; *COL1A1*, collagen type 1 alpha 1 chain; *COL3A1*, collagen type 3 alpha 1 chain.

Release of pentastatin from the basement membrane might also have patho(-physiological) effects on hPASMCs. Therefore, hPASMCs were treated with increasing concentrations of pentastatin for 24 h. Interestingly, pentastatin treatment increased the proliferation rate of hPASMCs, with a peak at 12.5 µg/mL (Fig. 5*A*). In contrast to hPAECs (Fig. 4*G*), no adverse effect on proliferation was observed with the highest concentration of pentastatin (50 µg/mL), suggesting different functional effects on hPAECs and hPASMCs (Fig. 5*A*). In addition to the increase in proliferation, vascular fibrosis contributes to the progression of PH (41, 42). Therefore, the effect of pentastatin on the expression of contractile versus synthetic genes was investigated and revealed increased expression of *COL1A1* and *COL3A1*, major fibrillar collagens involved in vascular fibrosis, following treatment with low concentrations (0.5 µg/mL) pentastatin (Fig. 5*B*).

No effect on proliferation (Supplemental Fig. S6, *A* and *B*) or expression of synthetic versus contractile genes (Supplemental Fig. S6*C*) was observed when hPASMCs were treated with the supernatant of pentastatin-activated hPAECs.

### Pentastatin Leads to an Increase in Mean Pulmonary Pressure and a Decrease in Vascular Integrity in an Ex Vivo Mouse Model

**Figure 6.**
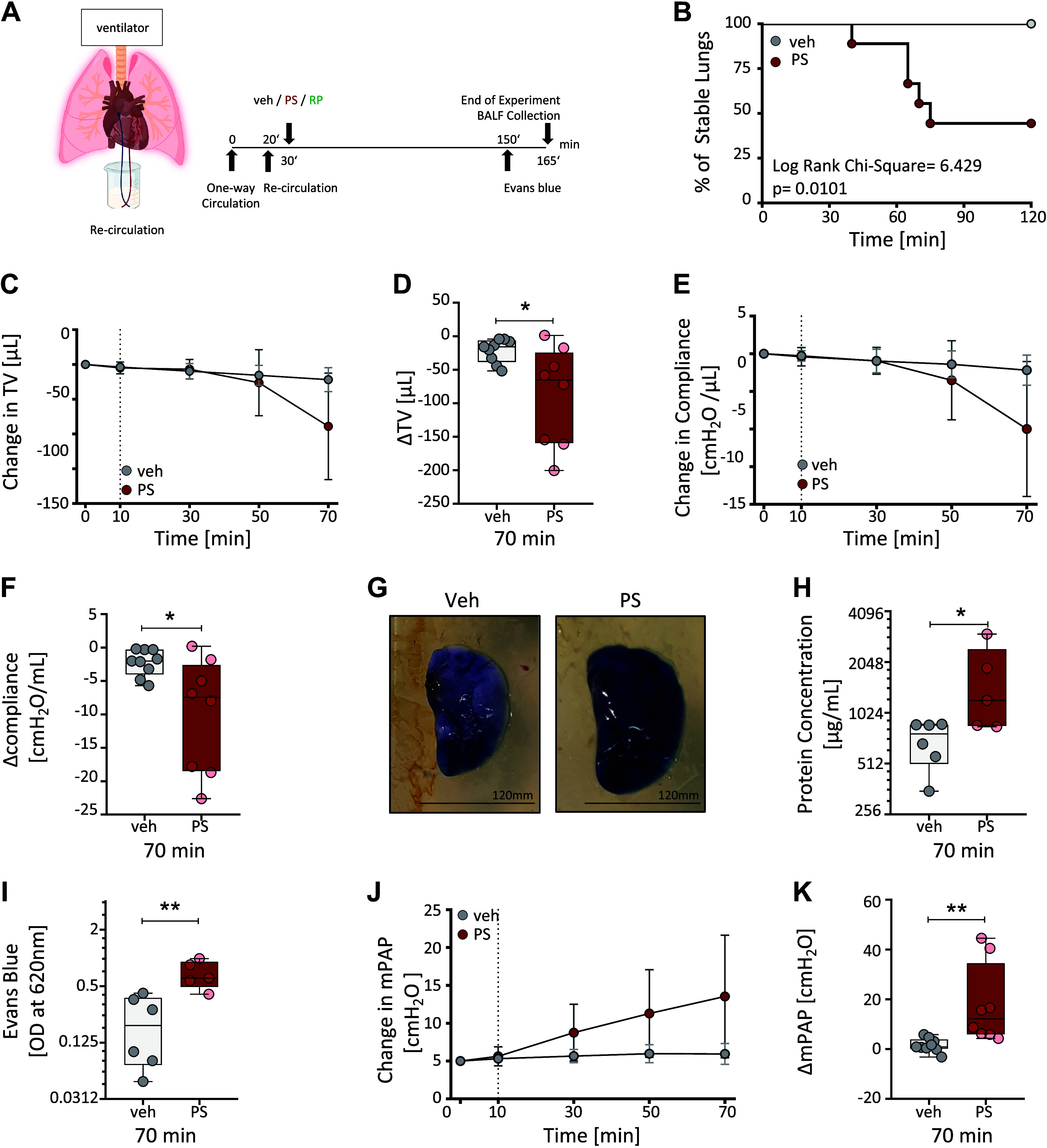
Pentastatin (PS) increases mean pulmonary arterial pressure (mPAP) in ex vivo isolated-perfused and ventilated lung mouse model (mIPL). *A*: schematic timeline of the PS treatment in ex vivo isolated-perfused and ventilated lung mouse model (mIPL). Mice lungs from 9- to 12-wk-old male wild-type C57BL/6J were used. Created with BioRender and published with permission. *B*: percentage of stable lungs throughout the 120 min of recirculation period represented by the Kaplan–Meier curve. *P* value <0.05; determined by Log-rank (Mantel–Cox) test. Real-time changes in lung physiology on the course of vehicle (veh, DMSO, *n* = 9) and PS (50 µg/mL, *n* = 9) treatment were determined by monitoring tidal volume (TV) (*C*) and compliance (*E*). Relative changes in TV (*D*) and compliance (*F*) at 70 min. *G*: representative pictures of perfused lungs at the end of the experiment. Scale bars: 120 mm. *H*: protein concentration in the circulation buffer. *Y*-axis set to log2 scale; Evans blue levels in bronchoalveolar lavage fluid (BALF) was measured at 620 nm presented (*I*). *Y*-axis set to log2 scale. *J*: real-time changes in mean pulmonary arterial pressure (mPAP). *K*: relative changes in mPAP at 70 min. veh vs. PS: *P < 0.05, **P < 0.01; determined by unpaired *t* test. Error bars represent the standard deviation (SD) in *C*, *E*, and *J*. DMSO, dimethyl sulfoxide.

We next used the isolated-perfused and ventilated lung mouse model to validate the biological relevance of pentastatin effects on the pulmonary vasculature ex vivo (Fig. 6*A*). Importantly, the pentastatin sequence displays a 100% identity between humans and mice, which enables the use of the same recombinant pentastatin peptide as used in in vitro studies. Notably, pentastatin preparation was free from LPS contamination (Supplemental Fig. S7). Isolated lungs were perfused with either vehicle, random peptide control (50 μg/mL), or pentastatin (50 μg/mL) for 120 min. Within this time frame, lungs perfused with vehicle (Fig. 6*B*) or random peptide control (Supplemental Fig. S8, *A*–*C*) were stably ventilated with minimal edema formation. In contrast, pentastatin-perfused lungs were significantly less stable as determined by severe worsening of the respiration to a point where tidal volume and compliance decreased by more than 60% compared with baseline (Fig. 6, *B*–*F*). Therefore, the effects of pentastatin were investigated for the first 70 min, which marked the turning point for lung stability. Over time, pentastatin treatment decreased the tidal volume (Fig. 6, *C* and *D*) and lung compliance (Fig. 6, *E* and *F*). In accordance with its effects on barrier disruption in vitro, pentastatin increased vascular permeability (Fig. 6*G*), as determined by increased protein concentrations in the circulating buffer (Fig. 6*H*) and extravasation of Evans blue in bronchoalveolar lavage fluid (BALF) after perfusion with pentastatin (Fig. 6*I*). Importantly, pentastatin led to an acute rise in mPAP in the isolated-perfused mouse lungs (Fig. 6, *J* and *K*).

**Figure 7.**
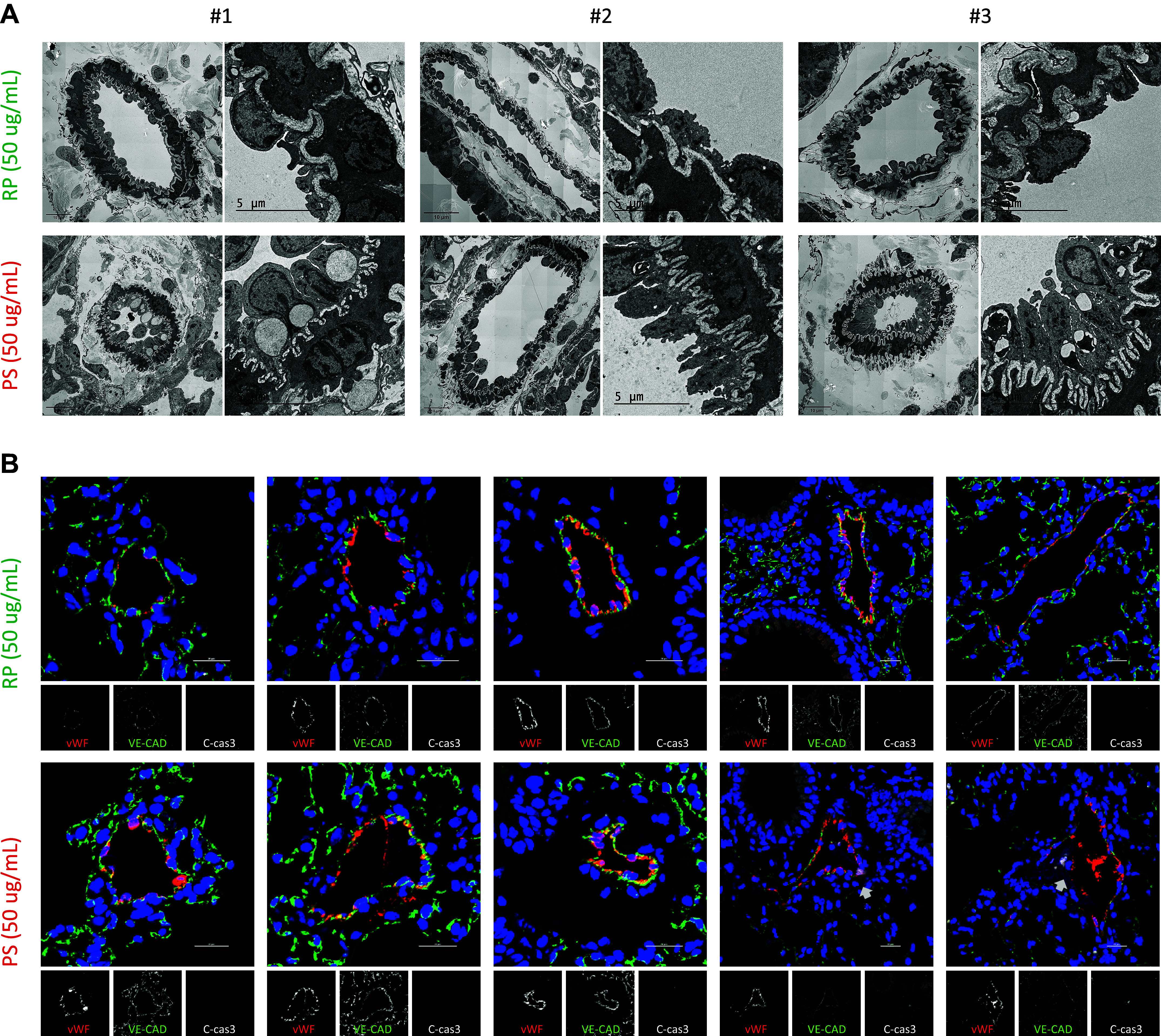
Pentastatin-perfused mouse lungs display an activated abnormal vascular endothelium. *A*: representative transmission electron micrographs of mouse lungs perfused either with random peptide (RP, 50 μg/mL) or pentastatin (PS, 50 μg/mL). Data are representative of *n* = 7 independent mouse lungs perfused either with RP (*n* = 3) or PS (*n* = 4). Scale bars: 10 μm and 5 μm, respectively. *B*: representative immunofluorescence images of mouse lungs perfused either with RP or PS. vWF is in red, VE-cadherin in green, cleaved caspase-3 in white (white arrows) and DAPI in blue. Images representative of *n* = 7 independent mouse lungs perfused either with RP (*n* = 3) or PS (*n* = 4). Scale bars: 20 μm. DAPI, 4′,6-diamidino-2-phenylindole dihydrochloride.

Next, to further investigate the effects of pentastatin on perfused lungs, PAs from those lungs were visualized at ultra- and microstructural levels using electron microscopy and immunofluorescence stainings. Ultrastructural analysis revealed a swollen, abnormal lysosome-rich pulmonary arterial endothelium in pentastatin-perfused lungs (Fig. 7*A*), which was accompanied by occasional endothelial cell death (Supplemental Fig. S8*D*). In addition, the recoiling of the pulmonary arterial elastica was observed in those lungs (Fig. 7*A*). Lungs perfused with random peptide control did not display any remarkable changes in the PAs at the ultrastructural level (Fig. 7*A* and Supplemental Fig. S8*D*). Immunofluorescence stainings supported the ultrastructural analysis, with an increased vWF signal within the PAs of pentastatin-perfused lungs, indicating an activated endothelium. In addition to that, loss of VE-cadherin and the appearance of cleaved caspase 3 positive cells were occasionally observed in some vessels (Fig. 7*B*).

### C-terminal NC1-ColIVα5 Fragments Are Found in Pulmonary Arteries and Plasma of Healthy Controls and Patients with IPAH

Finally, we aimed to validate the clinical relevance of our findings and sought to determine the localization of pentastatin on human tissue with vascular remodeling. Using an antibody raised against the NC1 domain of the ColIVα5 polypeptide (Gly1461 ∼ Thr1685), spanning the pentastatin sequence (Leu1516 ∼ Phe1535) (Supplemental Fig. S1, displaying proteolytic cleavage sites), we detected the presence of NC1- ColIVα5 fragments in pulmonary arteries of healthy donors and patients with end-stage IPAH (Supplemental Fig. S9*A*).

Investigation of human plasma from controls and patients with IPAH from our outpatient clinic (patients’ characteristics are found in Table 4) revealed tendentially increased levels of ColIVα5 and NC1-ColIVα5 fragments in IPAH (Supplemental Fig. S9*B*).

## DISCUSSION

Early endothelial injury, followed by the survival of hyper-resistant cells, is considered a major event in several lung diseases, including PH pathogenesis (43–46). Here, we identified that the C-terminal fragment of the basement membrane polypeptide collagen IVα5, termed NC1-ColIVα5, and its shorter peptide pentastatin, can contribute to disturbed vascular integrity by mediating endothelial activation, barrier disruption, and apoptosis. These cellular events were recapitulated in an ex vivo model where (ultra)structural changes in the endothelium were associated with edema formation and increased mean pulmonary arterial pressure.

Usually, BM-collagens are considered to be very stable proteins, however, in PH, increased expression and activity of proteases by both structural cells and a dysregulated immune cell profile likely contribute to their degradation, and subsequent liberation of novel bioactive fragments. In line, the C-terminal NC1- ColIVα5 domain harboring the pentastatin region contains several potential cleavage sites for proteases (Supplemental Fig. S1), which may result in its release. These include neutrophil elastase (NE, encoded by *ELANE)*, a protease previously described to be upregulated by neutrophils and SMCs in remodeled vessels of patients with PAH (7, 47). Subsequently, when using an antibody raised against the C-terminal NC1-ColIVα5/pentastatin domain, we detected its presence in the (neo-) intima and partially the media of remodeled PAs. Following proteolytic fragmentation, both PASMCs and PAECs may be exposed to high levels of pentastatin at the site of release. On both cell types, pentastatin-induced changes in cellular behavior that are clearly associated with the propagation of vascular remodeling. In hPASMCs, pentastatin increased proliferation, and the transcription of fibrillar collagens, such as *COL1A1* and *COL3A1*, shifted their phenotype towards a synthetic phenotype. Indeed, the expansion of synthetic and fibroblast-like cell states of SMCs accompanied by vessel fibrosis is a hallmark of vascular remodeling in PH (27, 41, 42). On hPAECs, pentastatin potently impaired endothelial barrier integrity. In line, recent advances have highlighted the significant role of endothelial cell dysfunction and vascular permeability in PH (31–33, 48–55). Various factors including increased flow rate (49), disturbed ion homeostasis (50–52), cytokines such as TNF-α (53–55), and growth factors such as VEGF (31–33), which induce endothelial cell barrier loss, have all been shown to cause or worsen PH.

The actions of pentastatin were not solely limited to endothelial barrier integrity but also extended directly to endothelial cell survival. Here, we identified pentastatin as a potent pro-apoptotic mediator for PAECs. Endothelial cell apoptosis is an initiating event in PH pathology and is considered to precede vascular remodeling and increased arterial pressure (43–46). This is supported by the fact that mice lacking a functional programmed cell death 4/caspase 3 axes in endothelial cells were protected against the development of PH (56). Similarly, pharmacological inhibition of apoptosis using a caspase inhibitor repressed PH onset and increased the expression of the intercellular junction protein PECAM-1 (57), a protein pivotal in maintaining endothelial barrier integrity (58, 59). Together, pentastatin may contribute to pulmonary vascular remodeling by disturbing several crucial functional processes of endothelial cells, such as barrier integrity and survival.

Previous studies on HUVECs suggested that pentastatin acts via β1 and/or β3-integrin subunits (17), however, the cognate receptor of NC1-ColIVα5/pentastatin has not been investigated previously in hPAECs. Here we showed a direct binding of the β1-integrin subunit to the NC1-ColIVα5 fragment and pentastatin, revealing the β1-integrin subunit as one of the functional receptors in hPAECs. Although integrin β1 subunit activation on hPAECs was clearly induced by pentastatin, pharmacological blocking of the β1 integrin subunit, only partially reduced the pentastatin-induced barrier disruption, suggesting additional involvement of other receptors. Interestingly, pentastatin displays a four amino acid homology (VCNF) with the bioactive T3 sequence of tum-5, the most potent anti-angiogenic region of the tum-5 sequence in tumstatin (13, 60). This region is known to exert its actions via αVβ3 integrins (13, 60, 61). Therefore, future studies should also investigate the involvement of αVβ3, as well as αVβ1 integrins in mediating the pentastatin-induced endothelial barrier dysfunction.

Integrins regulate cellular cues due to EC-ECM interactions. The functional relevance of integrin action has been established in experimental PAH (62, 63); however, so far, most studies have primarily focused on hPASMCs. On microvascular endothelial cells, the β1-integrin subunit regulates barrier function via VE-cadherin organization and, thus, plays a critical role in sustaining intact endothelial barrier and vessel integrity (37). Indeed, inhibition of the β1-integrin subunit partially limited the barrier disruption caused by pentastatin in our in vitro experiments. Increased expression of the receptor (β1-integrin) and enhanced levels of its ligand (NC1-ColIVα5/pentastatin) suggest a higher susceptibility of endothelial cells to pentastatin in IPAH. At present, increasingly specific integrin inhibitors are being developed and tested for their translational potential; PLN- 74809, a dual inhibitor for αVβ1 and αVβ6 integrins, is in Phase2a clinical trial with patients with pulmonary fibrosis (NCT04396756), which may open new avenues to clinically target pentastatin signaling.

Similarly, by inhibiting downstream signaling by Rho/ROCK, we partially reduced the pentastatin-induced endothelial barrier disruption. In line, previous studies have already identified RhoA/ROCK as a potential therapeutic target in PAH, as using ROCK inhibitors such as Y-27632 and Fasudil alleviated experimental PH (64, 65). Increased ROCK2 levels have been reported in patients with PAH, and ROCK activity has been associated with vascular SMC proliferation and contraction (66). In addition, mPAP and pulmonary vascular resistance of patients with IPAH declined when treated acutely with Fasudil (67). Together, this suggests therapeutic possibilities to indirectly inhibit pentastatin function, thereby improving endothelial cell function, and potentially ameliorating diseases such as PH. So far, options to target pentastatin directly are still hampered by its small size (2 kDa), and the current lack of either neutralizing antibodies or small molecule inhibitors.

Although this study adds further pieces to the understanding of the basement membrane and its components in pulmonary vascular pathology, there are still open questions to be addressed. At this stage, our data clearly identifies pentastatin as a driver of endothelial cell dysfunction. However, how these acute actions affect the development of chronic disease and whether endothelial injury precedes the release of pentastatin, or vice versa, is currently still unclear. As discussed earlier, changes in the inflammatory milieu and a pro-proteolytic environment during disease development suggest that the release of pentastatin would occur during the course of the disease. Here, repeated micro-injury to the endothelium together with the continuous proproliferative and profibrotic stimuli to SMCs would accumulate and further propagate the vicious cycle and, thus, vascular remodeling. Although our ex vivo animal model has a few limitations, such as the lack of systemic effects, or its short exposure time, it is possible to measure essential parameters involved in PH pathogenesis, such as pulmonary vascular resistance and pressure, in addition to respiratory mechanics and edema formation. Studies investigating the role of Dasatinib have already highlighted the translatability of this acute model to long-term PH settings (68–71). Future studies addressing the long-term effects of pentastatin will give further crucial information on its potential as a therapeutic target and highlight the physiologically role of endothelial cell injury in disease development and progression.

## DATA AVAILABILITY

Data will be made available upon reasonable request.

## SUPPLEMENTAL DATA

10.6084/m9.figshare.24037800 All Supplemental Material is available at: https://doi.org/10.6084/m9.figshare.24037800.

## GRANTS

F.V. was supported by the Austrian Research Promotion Agency (FFG, 874229) assigned to G.K. K.J. is supported by the start fund of the Medical University of Graz. L.M.M. is supported by the Austrian Science Fund (FWF, KLI 884-B). N.R. was supported by the Provincial Goverment of Styria through the PhD program RESPImmun. This research was funded partially by the Austrian Society for Pneumology (ÖGP, Wissenschaftsförderung 2022) assigned to A.C.M. This research was funded in part, by the Austrian Science Fund (FWF, DOC 129-B) and by the German Research Foundation (DFG, SFB 1213-Project-ID 268555672) assigned to G.K.

## DISCLOSURES

No conflicts of interest, financial or otherwise, are declared by the authors.

## AUTHOR CONTRIBUTIONS

A.C.M., K.J., and G.K. conceived and designed research; A.C.M., K.J., N.R., D.K., J.H. and M.W. performed experiments; A.C.M., K.J., F.V., D.K., V.F., and J.W. analyzed data; A.C.M., K.J., M.W., L.M.M., and G.K. interpreted results of experiments; A.C.M. prepared figures; A.C.M., G.K. and K.J. drafted manuscript; A.C.M., K.J., N.R., A.O., H.O., A.H., M.W., L.M.M., and G.K. edited and revised manuscript; A.C.M., K.J., N.R., F.V., D.K., J.H., V.F., J.W., P.M., K.H., A.O., H.O., A.H., M.W., L.M.M., and G.K. approved final version of manuscript.
